# Positive End-Expiratory Pressure (PEEP) Titration With Fraction of Inspired Oxygen (FiO₂)/PEEP Table and Esophageal Balloon Guidance in Obese Patients With Acute Respiratory Distress Syndrome: Description of Four Cases at 2600 m Above Sea Level

**DOI:** 10.7759/cureus.106612

**Published:** 2026-04-07

**Authors:** Anibal Cortes-Bravo, Guillermo Ortiz-Ruiz, Manuel Garay, Jonathan Alexander Guezguan-Perez, Cesar J Ortiz-Castellanos

**Affiliations:** 1 Critical Care Medicine, National Cancer Institute of Colombia (Instituto Nacional de Cancerología), Bogotá, COL; 2 Internal Medicine and Critical Care, Central-East Integrated Health Services Subnetwork (Subred Integrada de Servicios de Salud Centro Oriente), Bogotá, COL; 3 Critical Care Medicine, Hospital Santa Clara, Bogotá, COL; 4 Research, School of Medicine, Marista University of Mérida (Universidad Marista de Mérida), Mérida, MEX

**Keywords:** ards (acute respiratory distress syndrome), covid – 19, invasive mechanical ventilation (imv), obesity, positive end-expiratory pressure, prone position ventilation

## Abstract

The COVID-19 pandemic led to a global surge in acute respiratory distress syndrome (ARDS), frequently presenting with a phenotype characterized by preserved respiratory system compliance and low lung recruitability. While mechanical ventilation is essential for oxygenation, it can induce ventilator-induced lung injury (VILI) indistinguishable from ARDS. High-altitude environments (2,600 meters above sea level) and morbid obesity further complicate positive end-expiratory pressure (PEEP) titration. This study describes a personalized PEEP titration protocol guided by transpulmonary pressure (PTP) in obese patients with COVID-19-associated ARDS. We retrospectively analyzed a case series of four obese patients with severe ARDS managed in an intensive care unit. A PEEP titration protocol was implemented using esophageal pressure (Pes) monitoring to estimate PTP in both supine and prone positions. Oxygenation indices and lung mechanics were recorded. While transpulmonary driving pressure showed variable values, titrating PEEP to achieve an expiratory transpulmonary pressure (Ptpexp) close to 0 cmH2O revealed that lower PEEP levels were required during pronation to maintain alveolar stability, which coincided with significant improvements in oxygenation indices. Our findings suggest that PEEP should be personalized in obese patients with COVID-19-associated ARDS, particularly when managed at high altitudes. Esophageal manometry serves as a valuable tool to distinguish between lung and chest wall elastance, preventing overdistension and alveolar collapse. Although personalized titration optimized physiological variables, further research is needed to determine its impact on long-term clinical outcomes and mortality in this specific population.

## Introduction

The COVID-19 pandemic has resulted in a significant surge in cases of acute respiratory distress syndrome (ARDS). In 2016, a global study involving 459 intensive care units (ICUs) across 50 countries reported that 10% of ICU patients and 23% of those requiring mechanical ventilation met the criteria for ARDS [1,2]. In the context of COVID-19-associated ARDS, mortality trends have been observed that may be attributed to evolving changes in ventilatory management. An English cohort of 21,082 patients in 2020 showed mortality rates of 28.4% in high-dependency units and 32% in ICUs by the end of April. By June of the same year, these rates had decreased to 7.3% and 19.6%, respectively [3]. ARDS is a syndrome characterized by inflammatory pulmonary edema, severe hypoxemia, decreased lung compliance, and diffuse endothelial and epithelial injury [3,4]. It is associated with high mortality rates; Dennis et al. reported hospital mortality exceeding 30%, while trials involving moderate to severe ARDS have reported 90-day mortality rates as high as 43% [4,5].

Initially, COVID-19-associated ARDS was thought to follow the typical Berlin definition [6]. However, later descriptions by Gattinoni et al. identified a specific phenotype characterized by preserved respiratory system compliance and low lung recruitability [7,8]. Transpulmonary pressure (PTP) during spontaneous breathing increases due to a decrease in pleural pressure (Ppl) produced by the contraction of inspiratory muscles. During mechanical ventilation, PTP increases due to a rise in alveolar pressure (Palv) delivered by the ventilator. While mechanical ventilation is implemented to restore adequate oxygenation, research over the last two decades has shown that it can also induce or exacerbate lung damage indistinguishable from that caused by ARDS itself [8,9].

As a strategy to reduce atelectasis in ARDS, Ortiz et al. described the use of positive end-expiratory pressure (PEEP) [10]. PEEP aims to increase lung volume at the end of expiration, thereby decreasing deformability during inspiration and mitigating shear injury or atelectrauma [10-12]. One explanation for persistent mortality in ARDS may be related to the limitations of different PEEP and tidal volume titration strategies. Generally, these strategies reflect the mechanical behavior of the global respiratory system (lung and chest wall) rather than specific lung regions. This can generate disparate PTP values in areas with different mechanical conditions, potentially overdistending non-dependent lung areas while causing cyclic collapse and reopening of dependent areas [11,12].

The optimal method for titrating PEEP in patients with COVID-19-associated ARDS remains unknown. Randomized controlled trials have found that a strategy of higher versus lower PEEP did not significantly reduce mortality rates [13]. From a pulmonary mechanics perspective, PEEP titration seeks the ideal point on the pressure-volume curve to balance overdistension and alveolar collapse. Recommended PEEP levels for lung protection typically range between five and 24 cmH2O [13,14]. Although higher PEEP levels have shown protective effects in animal models of induced ARDS, clinical research in humans has not consistently demonstrated robust outcomes associated with these higher levels [13,15]. Amato et al. suggested that this discrepancy might occur because pleural pressure (Ppl) is often not accounted for, resulting in suboptimal PEEP application or the misinterpretation of high plateau pressures as indicators of overdistension [16].

Managing ARDS at 2,600 meters above sea level (m.a.s.l.) presents unique physiological challenges. At this altitude, the barometric pressure is approximately 560 mmHg compared to 760 mmHg at sea level. This reduction significantly lowers the inspired pressure of oxygen and alters gas density. Because standard fractional inspired oxygen concentration (FiO2)/PEEP tables, such as the ARDSnet high-PEEP protocol, were derived and validated at sea level, their direct application at high altitudes may lead to suboptimal PEEP titration. Consequently, oxygenation indices such as the partial pressure of arterial oxygen (PaO2)/FiO2 ratio must be interpreted with caution.

Furthermore, in morbidly obese patients, respiratory mechanics are further complicated by a massive increase in chest wall elastance. The weight of the abdomen and thoracic adipose tissue displaces the diaphragm, elevates baseline resting pleural pressure, and severely reduces functional residual capacity, promoting early dependent alveolar collapse. In this combined setting of obesity and high altitude, standard PEEP tables are often inadequate. Esophageal manometry provides a reliable surrogate for pleural pressure, allowing clinicians to calculate transpulmonary pressure (alveolar pressure minus pleural pressure). Without this bedside tool, it is impossible to distinguish whether an elevated plateau pressure reflects severe lung injury (low compliance) or simply the excessive mechanical load of the chest wall.

In this study, we retrospectively describe four obese patients with ARDS during the COVID-19 pandemic managed at 2,600 m.a.s.l. We utilized a PEEP trial guided by transpulmonary pressure, estimated through esophageal pressure monitoring in both supine and prone positions, to personalize ventilatory settings. This study aims to describe how personalized, PTP-guided PEEP differs from the baseline recommendations of the standard FiO2-high PEEP table in a population uniquely challenged by both morbid obesity and high-altitude physiology.

## Case presentation

We retrospectively reviewed a series of four morbidly obese patients admitted to the Intensive Care Unit (ICU) with severe COVID-19-associated ARDS at a high-altitude center (2,600 m.a.s.l.). All patients required invasive mechanical ventilation and management via a personalized PEEP titration protocol. Baseline clinical and demographic characteristics are detailed in Table 1, while initial ventilatory settings and advanced respiratory mechanics are summarized in Tables 2, 3, respectively.

**Table 1:** Baseline Clinical and Demographic Characteristics S. No.: serial number; BMI: body mass index; HTN: Hypertension; MI: myocardial infarction; HLP: hyperlipidemia; APACHE II: acute physiology and chronic health evaluation II.

S. No.	Patient	Age (years)	Gender	BMI (kg/m²)	Comorbidities	COVID-19 PCR	APACHE II	Outcome
1	1	57	Male	38.5 (Class II)	HTN, Cancer, HLP	Positive	30	Deceased
2	2	71	Male	34.2 (Class I)	HTN, MI, Smoking	Positive	32	Deceased
3	3	71	Female	42.1 (Class III)	HTN	Positive	27	Deceased
4	4	21	Female	36.8 (Class II)	Asthma, Seizures	Negative (Suspected)	28	Deceased

**Table 2:** Ventilatory Settings and Clinical Mechanics Data All measurements were performed under volume control assist-control (VC-AC) mode. Multiple rows for the same patient correspond to sequential measurement phases. FiO₂ was adjusted strictly to maintain SpO₂ ≥90%; in instances where it remained unchanged, patients had not met the physiological criteria for weaning. S. No.: serial number; VT: tidal volume; FiO₂: fraction of inspired oxygen; PEEP: positive end-expiratory pressure; SpO₂: peripheral oxygen saturation; P. Plat: plateau pressure; P. Esoph: esophageal pressure.

S. No.	Patient	Position	VT (mL)	FiO₂ (%)	PEEP (cmH₂O)	SpO₂ (%)	P. Plat (cmH₂O)	P. Esoph (cmH₂O)
1	1	Supine	440	70	16	92	36	16
2	1	Supine	440	70	20	90	42	17
3	1	Supine	440	70	10	94	29	15
4	2	Supine	420	70	20	90	43	19
5	2	Prone	420	50	15	92	26	18
6	3	Supine	416	70	16	90	34	18
7	3	Prone	398	50	15	92	26	14
8	4	Supine	380	55	14	90	43	23
9	4	Prone	350	50	14	92	36	20

**Table 3:** Advanced Respiratory Mechanics and Transpulmonary Pressures All measurements were performed under volume control assist-control (VC-AC) mode. Cells containing dashes (-) represent measurements that could not be reliably obtained retrospectively due to signal artifacts in the cardiogenic oscillation waveform. S. No.: serial number; DP: driving pressure; Ers: respiratory system elastance; Ecw: chest wall elastance; EL: lung elastance; Cdyn: dynamic compliance; Cstat: static compliance; PTP-DM: transpulmonary pressure direct method; PTP-E: expiratory transpulmonary pressure; PTPi-ED: inspiratory transpulmonary pressure derived from elastance; PTPe-ED: expiratory transpulmonary pressure derived from elastance; TPDP: transpulmonary driving pressure; DPPL: direct pleural pressure.

S. No.	Patient	DP (cmH2O)	Ers (cmH2O/L)	Ecw (cmH2O/L)	EL (cmH2O/L)	Cdyn (mL/cmH2O)	Cstat (mL/cmH2O)	PTP-DM (cmH2O)	PTP-E (cmH2O)	PTPi-ED (cmH2O)	PTPe-ED (cmH2O)	TPDP (cmH2O)	DPPL (cmH2O)
1	1	20.0	37.9	23.9	13.1	20.9	23.1	15.0	1.6	-	0.4	-0.4	24.5
2	1	-	-	-	-	-	-	17.0	1.8	-	-	-	-
3	1	-	-	-	-	-	-	16.0	1.1	-	-	-	-
4	2	22.0	41.9	20.9	21.0	18.2	19.0	19.0	-	21.05	9.9	19.8	22.4
5	2	16.0	30.9	16.9	14.0	26.25	26.25	14.0	0.5	20.6	10.9	3.0	17.2
6	3	18.0	33.9	44.9	11.0	14.0	22.0	17.0	0.4	11.0	21.1	16.6	59.6
7	3	11.0	25.9	38.9	13.0	16.5	36.1	14.0	0.6	12.0	13.0	13.4	58.0
8	4	30.3	44.2	31.6	12.6	10.4	12.5	12.4	-	12.6	10.0	30.7	36.0
9	4	22.0	35.9	27.5	12.6	11.2	17.2	8.5	-1.4	12.6	10.0	36.0	36.0

This series includes two typical cases of COVID-19-associated ARDS (cases one and three) and two complex, atypical cases (cases two and four). While case two presented with obstructive shock secondary to a massive pulmonary embolism, and case four with post-cardiac arrest aspiration pneumonia [Reverse Transcription Polymerase Chain Reaction (RT-PCR) negative], both shared the profound mechanical phenotype of a "small lung" aggravated by morbid obesity at high altitude. This common mechanical challenge justifies their inclusion to demonstrate the utility of personalized PEEP titration in preventing cyclic alveolar collapse. Formal lung recruitability (e.g., recruitment-to-inflation ratio) was not assessed before balloon insertion, relying instead on continuous physiological feedback. The cohort included three older adults (57-71 years) and one 21-year-old. All four met the criteria for obesity, body mass index (BMI) >30 kg/m², and presented with critical respiratory failure upon admission.

Case one

A 57-year-old male of mixed ethnicity presented to the emergency department with an eight-day history of worsening lower back pain and lower limb edema. His medical history was significant for morbid obesity (BMI 38.5 kg/m², Class II), hypertension, hypercholesterolemia, and prostate cancer (status post-prostatectomy). Clinical evaluation revealed thoracoabdominal dissociation and crepitus across all lung quadrants. He rapidly deteriorated into mixed ventilatory failure and pulmonary shock, requiring ICU transfer. On admission, he was sedated [Richmond Agitation-Sedation Scale (RASS)-2] [17] and required vasopressor support with norepinephrine (0.1 mcg/kg/min). Laboratory findings confirmed a severe acute respiratory syndrome coronavirus 2 (SARS-CoV-2) positive RT-PCR, leukocytosis, elevated C-reactive protein (CRP), and high D-dimer/ lactate dehydrogenase (LDH) levels. Chest radiography showed diffuse involvement in all four quadrants, and his admission acute physiology and chronic health evaluation II (APACHE II) score was 30 [18]. His initial PaO2/FiO2 ratio was 85. Following 24 hours of mechanical ventilation without oxygenation improvement, an esophageal balloon was inserted. Guided by transpulmonary pressure [19, 20], his PaO2/FiO2 increased significantly to 197, allowing for the withdrawal of neuromuscular relaxation and vasopressors. However, his clinical course was later complicated by cholestatic hyperbilirubinemia and septic abdominal shock. Despite resuscitation efforts following cardiac arrest (pulseless electrical activity), the patient did not survive.

Case two

A 71-year-old male smoker with a history of hypertension and a prior myocardial infarction (stented four years earlier) presented with a six-day history of non-productive cough and progressive dyspnea. He presented with Class I obesity (BMI 34.2 kg/m²). On admission, he was in critical condition with an oxygen saturation of 40% on room air and a Glasgow Coma Scale (GCS) of 7/15 [21]. Initial management with a non-rebreather mask improved his GCS to 11/15 [21], but severe respiratory failure necessitated immediate intubation. His initial PaO2/FiO2 ratio was 68, and his admission APACHE II score was 32. An electrocardiogram revealed an S1Q3T3 pattern, and the clinical picture progressed to obstructive shock [22] secondary to a massive pulmonary embolism. After 12 hours of mechanical ventilation, an esophageal balloon was inserted due to worsening mechanics. The patient’s ICU stay was remarkably complex; coronary angiography showed healthy epicardial arteries, leading to a diagnosis of viral myocarditis. His course was further complicated by acute kidney injury, de novo obstructive biliary syndrome, and gastrointestinal bleeding from large esophageal varices discovered during endoscopy. A diagnosis of cirrhosis and suspected Budd-Chiari syndrome was eventually made as his multi-organ function continued to decline until his death.

Case three

A 71-year-old female homemaker of mixed ethnicity with morbid obesity (BMI 42.1 kg/m², Class III) and hypertension presented with a ten-day history of asthenia, fever, and progressive functional decline, New York Heart Association (NYHA) II/IV. On admission, she was hypotensive with a mean arterial pressure (MAP) 57.7 mm Hg) and tachycardic, requiring sedation (RASS -2) [17] and protective mechanical ventilation for SARS-CoV-2 pneumonia. Her initial PaO2/FiO2 ratio was 72. Laboratory tests indicated severe oxygenation impairment and elevated inflammatory markers (CRP, LDH, D-dimer). Her chest X-ray showed diffuse reticulonodular infiltrates, and her admission APACHE II score was 27 [18]. After 18 hours of failing to improve on standard ventilatory settings, transpulmonary pressure was calculated using an esophageal balloon [19, 20] in both supine and prone positions. Within 12 hours of this personalized titration, her PaO2/FiO2 improved to 147. She was successfully liberated from mechanical ventilation four days later and transferred to general hospitalization. However, on day 26 of her stay, she developed a right middle cerebral artery stroke with left hemiparesis. Following a family decision to limit therapeutic efforts due to her significant functional limitations, she suffered new ventilatory failure and passed away.

Case four

A 21-year-old female with a history of mixed ethnicity, asthma, and seizures presented with generalized tonic-clonic seizures and a dry cough. She had morbid obesity with a BMI of 36.8 kg/m² (Class II). Having received only a single dose of the SARS-CoV-2 vaccine, she was initially intubated at a primary hospital [23] with a GCS of 7/15 [21]. During transfer to the ICU, she suffered a cardiorespiratory arrest due to ventilatory uncoupling and a misplaced orotracheal tube. Spontaneous circulation was restored after eight minutes of resuscitation. She was subsequently managed for post-resuscitation status, aspiration pneumonia, and status epilepticus. Her admission APACHE II score was 28, and her initial PaO2/FiO2 ratio was 65. Although her RT-PCR for COVID-19 was negative, she was treated for secondary COVID-19-associated ARDS due to her recent vaccination history and suggestive respiratory symptoms. An esophageal balloon was inserted 12 hours post-intubation to manage elevated airway pressures. Her ICU course was marked by severe anemia (Hb 4 g/dL) and a secondary infection with Acinetobacter baumanii KPC isolated from blood cultures. Neurological monitoring confirmed severe and irreversible hypoxic-ischemic encephalopathy. Months after admission, following a decision to limit therapeutic efforts, the patient passed away.

Implementation of the PEEP titration protocol

To optimize ventilatory support, an esophageal balloon was inserted in all patients to measure esophageal pressure (Pes) as a proxy for pleural pressure. This facilitated the calculation of transpulmonary pressure (PTP) for personalized PEEP titration, moving beyond conventional Berlin definition management [6,17]. The step-by-step protocol for esophageal balloon insertion and the subsequent PEEP titration logic are illustrated in Table 4.

**Table 4:** Checklist for Esophageal Balloon Catheter Insertion and PEEP Titration Paw: Airway pressure; Pes: Esophageal pressure; Ptpexp: Expiratory transpulmonary pressure; RASS: Richmond Agitation-Sedation Scale. Protocol developed at Hospital Santa Clara by Dr. Aníbal Cortés Bravo.

Item No.	Checklist
1	Ventilator configuration
1a	Use a Mindray SV800 mechanical ventilator equipped with an auxiliary port dedicated to esophageal pressure monitoring
1b	Set lung-protective volume-controlled ventilation according to ARDS guidelines
2	Patient preparation
2a	Induce deep sedation and neuromuscular blockade to achieve a Richmond Agitation–Sedation Scale (RASS) score of −4 to -5
2b	Apply topical airway anesthesia using lidocaine gel (nasal insertion) or lidocaine spray (oral insertion)
3	Esophageal balloon catheter insertion
3a	Advance the esophageal balloon catheter to 60 cm from the incisors to confirm intragastric placement, then withdraw to 40 cm to obtain esophageal pressure (Pes)
3b	Perform a balloon leak test by pre-inflating the balloon to determine the minimal inflation volume required for accurate pressure transmission
4	Monitor calibration and setup
4a	Zero-reference esophageal pressure via the PAUX interface on the Mindray SV800 ventilator
4b	Connect the catheter to PAUX 1 and activate continuous Pes monitoring
5	Balloon inflation and signal optimization
5a	Inflate the balloon with manufacturer-recommended volume (1 mL for Cooper Surgical; 4 mL for Nutrivent catheter)
5b	Gradually withdraw air until 1–1.5 mL remains, optimizing waveform morphology and minimizing artifacts
6	Verification of gastric positioning
6a	Apply gentle manual abdominal compression (≈2 cm displacement)
6b	Confirm gastric placement by observing a positive Pes deflection without a concomitant change in airway pressure (Paw)
7	Final esophageal positioning
7a	Slowly withdraw the catheter until cardiogenic oscillations appear on the Pes waveform, indicating mid-esophageal positioning
7b	Secure the catheter at the nostril once stable and reproducible signals are confirmed
7c	Record baseline Pes values
8	Occlusion maneuver for validation
8a	Perform an end-expiratory airway occlusion maneuver (“Expiratory Hold”)
8b	Apply gentle anterior chest wall compression (≈2 cm displacement)
9	Physiological validation of catheter position
9a	Measure changes in airway pressure (ΔPaw) and esophageal pressure (ΔPes) during compression
9b	Confirm correct positioning when the ΔPaw/ΔPes ratio ranges between 0.8 and 1.2 [20]
10	Transpulmonary pressure–guided PEEP titration
10a	Confirm inspiratory transpulmonary pressure (Ptp,ins) between 17 and 21 cmH₂O
10b	Confirm expiratory transpulmonary pressure (Ptp,exp) between −1 and +1 cmH₂O
10c	In the supine position, titrate PEEP downward from the baseline FiO₂/PEEP table value until Ptp,exp approaches zero
10d	Adjust FiO₂ to maintain peripheral oxygen saturation ≥90% and obtain arterial and venous blood gases to calculate PaO₂/FiO₂
10e	In the prone position, retitrate PEEP to maintain near-neutral Ptp,exp
10f	Adjust FiO₂ to maintain oxygen saturation ≥90% and reassess PaO₂/FiO₂
10g	Maintain ventilatory settings throughout the prone cycle for 16–18 hours
10h	Reestablish ventilatory parameters derived from the supine position upon completion of the prone cycle

Strict adherence to the clinical protocol required deep sedation and neuromuscular blockade, targeting a RASS score between -4 and -5. Once the catheter was in place, we calculated key respiratory mechanics-including driving pressure (DP), respiratory system elastance (ELrs), and lung elastance (EL). Our PEEP titration aimed for an expiratory transpulmonary pressure (Ptpexp) near 0 cm H2O, with a specific emphasis on monitoring physiological shifts during prone positioning. The detailed formulas and definitions used for these ventilatory variables are presented in Table 5.

**Table 5:** Formulas and Definitions for Ventilation Mechanics S. No.: Serial number; Pplat: Plateau pressure; PEEP: Positive end-expiratory pressure; VT: Tidal volume; Pes: Esophageal pressure; Ptp: Transpulmonary pressure; Paw: Airway pressure.

S. No.	Variable	Abbreviation	Formula
1	Driving pressure	DP	Pplat - PEEP
2	Respiratory system elastance	ELrs	(Pplat - total PEEP) / VT
3	Chest wall elastance	ELcw	(Inspiratory Pes - Expiratory Pes) / VT
4	Respiratory system elastance: Talmor et al. method [14]	ELrsT	((Pplat - PEEP) / VT) * ELcw
5	Chest wall elastance: Talmor et al. method [14]	ELcwT	(Inspiratory Pes - Expiratory Pes) / VT
6	Pulmonary elastance	ELL	Ers - Ecw
7	Dynamic compliance	Cdyn	VT / (Peak pressure - PEEP)
8	Static compliance	Cstat	VT / (Pplat - PEEP)
9	Transpulmonary pressure (direct method)	Ptp ins	Pplat - Inspiratory Pes
10	Transpulmonary driving pressure	TPDP	Inspiratory Ptp - Expiratory Ptp
11	Pleural pressure	Ppl	(Paw * ELcw) / ELrs

In all four cases, continuous monitoring revealed significant discrepancies between standard table recommendations and physiological needs. Transitioning from table-based PEEP to PTP-guided PEEP resulted in observable changes in oxygenation. For instance, following personalized titration and prone positioning, PaO2/FiO2 improved from 85 to 197 in Case 1 and from 72 to 147 in Case 3. Analysis of advanced mechanics highlighted the extreme physiological derangements in this cohort. Notably, Case 3 exhibited a profoundly elevated estimated direct pleural pressure (DPPL of 59.6 cmH2O) in the supine position, underscoring the severe impact of Class III obesity on chest wall mechanics. Furthermore, Case 4 demonstrated abnormally high transpulmonary driving pressures (TPDP), likely reflecting the heterogeneous nature of her lung injury (aspiration pneumonia and anoxic injury) compared to the viral ARDS seen in the other patients.

Clinical Evolution and Final Outcomes

While PTP-guided ventilation optimized these mechanics, severe comorbidities and multiorgan failure ultimately dominated the clinical course. All four patients eventually passed away due to complications such as septic shock, myocardial infarction, or irreversible encephalopathy. In each case, PEEP personalization allowed for a unique assessment of lung mechanics, even though it did not alter the final outcome.

Informed consent was obtained from the next of kin of all four patients presented in this case series for the publication of their clinical data. This study was conducted in accordance with the ethical standards of the institutional research committee (Instituto Nacional de Cancerología) and the Helsinki Declaration.

## Discussion

This case report documents four cases observed during the most critical phase of the COVID-19 pandemic. While we implemented a personalized PEEP titration strategy, the clinical reality was devastating: all four patients eventually succumbed to their illness. This high mortality is not an isolated finding; in resource-constrained settings, ICU mortality for severe ARDS can reach a staggering 70% [23]. To manage these complex shock states, we relied on continuous lactate monitoring as a cornerstone for assessing tissue oxygenation, given its strong predictive value for poor outcomes in critical care [22].

Our initial approach followed standard lung-protective ventilation and the traditional FiO2/PEEP tables. However, as plateau pressures rose-frequently reflecting an increase in chest wall elastance rather than lung injury alone-we transitioned to the institutional esophageal balloon protocol [10, 19]. Esophageal manometry remains the only bedside tool that allows us to distinguish between airway pressure and the pressure exerted by the chest wall, specifically providing an estimate of pleural pressure [14].

By monitoring transpulmonary pressure in real-time, we adjusted PEEP to maintain a slightly positive expiratory transpulmonary pressure (Ptpexp). The physiological goal was simple yet ambitious: keep recruitable alveolar units open to prevent the cycle of collapse and reopening known as atelectrauma [9, 14, 19]. Interestingly, our observations in this series suggest that prone positioning facilitates a more even distribution of alveolar inflation, which in turn levels the Ptpexp and makes it less negative [8]. By targeting a Ptpexp near zero, we found that patients often required lower PEEP levels during pronation while simultaneously showing improved oxygenation. However, it is critical to note that this was not a universal finding; as observed in Case 4, PEEP requirements remained unchanged (14 cmH2O) between supine and prone positions, underscoring the need for continuous, individualized reassessment rather than applying blanket physiological assumptions.

These findings echo strategies validated in trials like EPVent, which champion an "open lung" approach by ensuring end-expiratory transpulmonary pressure remains positive [14, 20, 19]. In our cohort, the morbid obesity of the patients was a dominant factor; their increased chest wall elastance often pushed the traditional safety limits of mechanical ventilation. Furthermore, managing these patients at 2,600 m.a.s.l. (barometric pressure of ~560 mmHg) likely rendered standard sea-level FiO2/PEEP tables inadequate, as lower ambient pressures alter baseline oxygenation indices. We observed that compliance was tied directly to ARDS severity, yet Ptp remained remarkably stable-between 1 and -1 cm H2O-once patients were pronated.

Limitations and Future Perspectives 

The clinical use of esophageal manometry is not without controversy. Discrepancies in transpulmonary pressure estimates have led some questions as to how accurately it reflects local distending pressure [7, 8, 14]. Moreover, the specialized equipment required is often unavailable in low-resource ICUs. Our study provides a physiological snapshot of lung mechanics at high altitude, but its retrospective design and small sample size mean we cannot draw broad conclusions about mortality or clinical outcomes. We must be explicit that our conclusions concern short-term physiological and mechanical optimization only.

A significant limitation of this retrospective analysis is the absence of complete, stepwise arterial blood gas (ABG) data (pH, PaCO2) for every titration stage, as clinical priority was given to oxygenation indices (SpO2 and PaO2/FiO2) during the pandemic's peak. Notably, Case 3 demonstrates the potential of this approach: PEEP personalization allowed for successful lung recruitment and liberation from mechanical ventilation. Her ultimate death was due to a subsequent neurological complication (stroke) rather than primary refractory respiratory failure. In contrast, Cases 1 and 2 succumbed to septic and obstructive shock, and Case 4 to irreversible hypoxic-ischemic encephalopathy. We view these results as a call for more robust, prospective research to test these physiological hypotheses in larger cohorts.

## Conclusions

The titration of PEEP guided by esophageal balloon-derived expiratory transpulmonary pressure (Ptpexp) allows for the personalization of ventilatory settings in patients with severe ARDS. This approach is particularly critical for obese patients managed at an altitude of 2,600 m.a.s.l., where altered barometric pressure and massive chest wall elastance present unique mechanical challenges that confound standard FiO2/PEEP tables. Our findings indicate that esophageal manometry provides a valuable bedside tool for distinguishing between lung and chest wall elastance, enabling more precise, individualized ventilatory adjustments.

While this series demonstrates the feasibility of physiological optimization, its retrospective nature, the lack of systematic ABG reporting, and the ultimate mortality of the patients driven largely by extra-pulmonary complications limit conclusions regarding survival. Future prospective trials are required to confirm if transpulmonary pressure-guided titration leads to superior clinical results compared to standard PEEP strategies in this specific population.

## References

[1] Bellani G, Laffey JG, Pham T (2016). Epidemiology, patterns of care, and mortality for patients with acute respiratory distress syndrome in intensive care units in 50 countries. JAMA.

[2] Meyer NJ, Gattinoni L, Calfee CS (2021). Acute respiratory distress syndrome. Lancet.

[3] Dennis JM, McGovern AP, Vollmer SJ, Mateen BA (2021). Improving survival of critical care patients with coronavirus disease 2019 in England: a national cohort study, March to June 2020. Crit Care Med.

[4] Meade MO, Cook DJ, Guyatt GH (2008). Ventilation strategy using low tidal volumes, recruitment maneuvers, and high positive end-expiratory pressure for acute lung injury and acute respiratory distress syndrome: a randomized controlled trial. JAMA.

[5] Thompson BT, Chambers RC, Liu KD (2017). Acute respiratory distress syndrome. N Engl J Med.

[6] Ranieri VM, Rubenfeld GD, Thompson BT (2012). Acute respiratory distress syndrome: the Berlin Definition. JAMA.

[7] Marini JJ, Gattinoni L (2020). Management of COVID-19 respiratory distress. JAMA.

[8] Gattinoni L, Chiumello D, Caironi P, Busana M, Romitti F, Brazzi L, Camporota L (2020). COVID-19 pneumonia: different respiratory treatments for different phenotypes?. Intensive Care Med.

[9] Chiumello D, Cressoni M, Colombo A (2014). The assessment of transpulmonary pressure in mechanically ventilated ARDS patients. Intensive Care Med.

[10] Ortiz-Ruiz G, Dueñas-Castel C, Garay-Fernández M (2022). Utilidad de la medición de presión esofágica en la ventilación mecánica: individualizando las variables fisiológicas. Acta Colomb Cuid Intensivo.

[11] Dreyfuss D, Saumon G (1998). Ventilator-induced lung injury: lessons from experimental studies. Am J Respir Crit Care Med.

[12] Gattinoni L, Marini JJ, Pesenti A, Quintel M, Mancebo J, Brochard L (2016). The "baby lung" became an adult. Intensive Care Med.

[13] Briel M, Meade M, Mercat A (2010). Higher vs lower positive end-expiratory pressure in patients with acute lung injury and acute respiratory distress syndrome: systematic review and meta-analysis. JAMA.

[14] Talmor D, Sarge T, Malhotra A (2008). Mechanical ventilation guided by esophageal pressure in acute lung injury. N Engl J Med.

[15] Tremblay L, Valenza F, Ribeiro SP, Li J, Slutsky AS (1997). Injurious ventilatory strategies increase cytokines and c-fos m-RNA expression in an isolated rat lung model. J Clin Invest.

[16] Amato MB, Barbas CS, Medeiros DM (1998). Effect of a protective-ventilation strategy on mortality in the acute respiratory distress syndrome. N Engl J Med.

[17] Sessler CN, Gosnell MS, Grap MJ (2002). The Richmond Agitation-Sedation Scale: validity and reliability in adult intensive care unit patients. Am J Respir Crit Care Med.

[18] Knaus WA, Draper EA, Wagner DP, Zimmerman JE (1985). APACHE II: a severity of disease classification system. Crit Care Med.

[19] Sarge T, Baedorf-Kassis E, Banner-Goodspeed V (2021). Effect of esophageal pressure-guided positive end-expiratory pressure on survival from acute respiratory distress syndrome: a risk-based and mechanistic reanalysis of the epvent-2 trial. Am J Respir Crit Care Med.

[20] Akoumianaki E, Maggiore SM, Valenza F (2014). The application of esophageal pressure measurement in patients with respiratory failure. Am J Respir Crit Care Med.

[21] Teasdale G, Jennett B (1974). Assessment of coma and impaired consciousness. A practical scale. Lancet.

[22] Kompanje EJ, Jansen TC, van der Hoven B, Bakker J (2007). The first demonstration of lactic acid in human blood in shock by Johann Joseph Scherer (1814-1869) in January 1843. Intensive Care Med.

[23] Tumukunde J, Sendagire C, Ttendo SS (2019). Development of intensive care in low-resource regions. Curr Anesthesiol Rep.

